# Randomised trial of wide-field guided PRP for diabetic macular oedema treated with ranibizumab

**DOI:** 10.1038/s41433-019-0342-1

**Published:** 2019-02-06

**Authors:** S. James Talks, Devangna Bhatia, Geeta Menon, Abosede Cole, Haralabos Eleftheriadis, Louise Downey, Ngai Victor Chong, Sobha Sivaprasad, on behalf of the RDP study group

**Affiliations:** 1 Newcastle Eye Centre, Newcastle Upon Tyne NHS Hospitals Foundation Trust, Tyne and Wear, UK; 2 Frimley Park Hospitals NHS Foundation Trust, Camberley, GU16 7UJ UK; 3 University Hospitals Bristol NHS Foundation Trust, Bristol, BS2 8AE UK; 4 King’s College Hospital NHS Foundation Trust, London, SE5 9RS UK; 5 Hull Royal Infirmary, Hull and East Yorkshire Hospitals NHS Trust, Hull, HU3 2JZ UK; 6 Oxford Eye Hospital, Oxford University Hospitals NHS Foundation Trust, Oxford, OX3 9DU UK; 7 Moorfields Eye Hospital NHS Foundation Trust, London, EC1 UK

**Keywords:** Outcomes research, Eye diseases, Diabetes complications

## Abstract

**Background:**

Diabetic macular oedema (DMO) is effectively treated with ranibizumab but multiple injections are required. Where there is also peripheral ischaemia, it has been promoted that targeted panretinal photocoagulation (PRP) may reduce the number of injections.

**Method:**

Patients with optical coherence tomography confirmed DMO and Ultra-widefield Fundus Fluorescein Angiography confirmed peripheral retinal ischaemia were randomised to PRP plus ranibizumab or ranibizumab monotherapy. After three injections, repeat injections were given until the visual acuity was stable and the macula was dry. Re-treatment was given if there was a drop of visual acuity and/or a recurrence of intra-retinal fluid. The primary outcome was the number of repeat injections required after the first 6 months up until 1 year.

**Results:**

There were 49 patients, 25 in the ranibizumab only group and 24 in the ranibizumab + PRP group recruited at seven UK sites. The average number of injections in the ranibizumab-only arm was 6.84 over 1 year and 2.52 between months 6 and 12. The average number of injections in the combined arm was 6.67, with the number of injections in the second 6 months 1.92. For the primary outcome, comparing the number of 6- to 12-month injections, the result was not statistically significant (*p* = 0.33).

**Conclusion:**

The addition of targeted PRP to areas of non-perfusion in a patient with DMO does not reduce the number of injections required in the first year. It seems most likely that local VEGF at the macula is the main cause of DMO.

## Introduction

Although centre-involving diabetic macular oedema (DMO) is effectively treated by anti-vascular endothelium growth factor (VEGF) injections, many patients require a large number of injections, at least in the first year. In the DRCR.net study (Protocol I) [1], a median of eight injections were required in the first year in the two ranibizumab arms. In the Restore study [2], a mean of seven injections were given in the first year. These treatments are expensive and cause a significant burden to the patient, their caregivers and the healthcare system.

The exact stimulus for VEGF production in DMO is unclear. In eyes with DMO and ischaemic peripheral retina [3], it has been postulated that the VEGF drive may be from the peripheral retina. Therefore, panretinal photocoagulation (PRP) targeted to peripheral non-perfused retina may reduce the VEGF load and in turn reduce the number of anti-VEGF injections required.

A recently published small trial, the DAVE study [4], did not find a reduction in the number of injections required over 3 years. This study evaluated 40 eyes of 29 patients and performed several sessions of PRP to ensure all the ischaemic areas of retina were treated. PRP itself can induce macular oedema and so possibly too much laser counteracted the potential benefit. Indeed, in this study there was less reduction of central retinal thickness (CRT) in the combined injection/PRP arm compared with monotherapy, 302 µm compared with 152 µm (*p* = 0.03). The RDP study used a standard amount of laser targeted to the ischaemic area of peripheral retina, imaged by Ultra-widefield Fundus Fluorescein Angiography (UWFFA), as a one-off treatment to assess if the number of injections in the second 6 months of the first year of treatment was reduced. The second 6 months was pre-specified to make allowance for any possible initial worsening of the DMO due to the laser.

## Method

The RDP study was a multicentre UK-based study run between 2014 and 2017. Patients with optical coherence tomography (OCT) (Heidelberg Engineering, Heidelberg, Germany) confirmed centre-involving DMO and UWFFA (Optomap P200, Optos PLC, Dunfermline, United Kingdom) confirmed peripheral retinal ischaemia were randomised to PRP plus ranibizumab or ranibizumab monotherapy.

Prior to any study-related procedures, all patients gave informed consent. The study had ethics approval (REC reference 13/NE/0197; IRAS 121940) and was registered with the clinical trial register (ISRCTN84503751). The study was performed in line with Good Clinical Practice in research guidelines and conformed with those of the Declaration of Helsinki.

The inclusion and exclusion criteria used are shown in Table 1. Eyes with new vessels were excluded. The assessment of the OCT scans and fundus fluorecein angiogram (FFA) was performed by the investigators to confirm eligibility. The images were subsequently sent to a reading centre.

**Table 1:** Key inclusion and exclusion criteria

Inclusion criteria	Exclusion criteria
Visual acuity 20/32 (80 EDTRS letters at 4 m) to 20/320 (30 EDTRS letters 4 m chart)	visual acuity worse than 20/320
Macular oedema secondary to diabetic retinopathy. (OCT thickness of > 300 µm central subfield, on spectral domain OCT (Spectralis Heidelberg engineering)	Rubeosis
Peripheral ischaemia seen on UWFFA (20% of the peripheral retina or more)	Proliferative diabetic retinopathy
Patient able to give consent and take part in all study procedures	No recent change to antihypertensive treatment within 2 months of start of study
	BP >180/110 mmHg (if elevated, treatment may be modified, and patients may subsequently be considered for recruitment after at least 2 months on new treatment regime)
	Unable to give consent or take part in all study procedures
	Other conditions that might interfere with the assessment of the eye such as cataract or prevent the macular oedema from settling such as vitreomacular traction, epiretinal membrane, to a degree that would in the opinion of the investigator affect response to treatment; conditions that would prevent the visual acuity improving such as foveal atrophy, uveitis
	Previous macular laser within 4 months in the study eye
	Previous peripheral (PRP) laser in the study eye
	Previous injection therapy within last 6 months in the study eye
	Pregnant
	Uncontrolled systemic illness that in the opinion of the investigator would preclude involvement
	Systemic steroid treatment within 3 months of recruitment, or during the study
	Cataract or other intraocular surgery within 3 months
	Previous vitrectomy

*ETDRS* Early Treatment Diabetic Retinopathy Study, *OCT* optical coherence tomography, *UWFFA* Ultra-widefield Fundus Fluorescein Angiography, *BP* blood pressure, *PRP* panretinal photocoagulation

Randomisation was by patients rather than eyes, as one eye per patient was included in the study. Which eye was chosen, if both were eligible, was at the investigator’s discretion. Equal numbers of patients were randomised either to receive or not to receive PRP. Randomisation was performed via the randomisation service Sealed Envelope.

The ranibizumab treatment criteria were: three injections 28 days apart in all cases ( ± 5 days) then repeat injections until two stable BCVA measures (within five LogMAR letters) and the macula was dry as judged by the investigator. Re-treatment was given if there was a drop of visual acuity of >5 letters, in the opinion of the investigator due to a recurrence of DMO, or a recurrence of intra-retinal fluid, as judged by the examining doctor. All patients were given three monthly ranibizumab injections and then followed up at monthly intervals with repeat visual acuity testing and OCT for a year. Repeat ranibizumab was given based on the specified re-treatment criteria.

No macular laser was planned but if the physician felt it was in the best interests of the patient to have some laser then it could have been applied but only after 6 months. No additional PRP was planned unless the patient developed rubeosis or new vessels that were not controlled by the injections.

### Intervention

The intervention (PRP) arm was given 2000, 200 micrometre, laser spots with the Pascal laser (Topcon (GB), Newbury, United Kingdom), 2 weeks after the first ranibizumab injection, to the area of ischaemia seen on UWFFA. An UWF (Ultra-widefield) colour and UWFFA was repeated at 1 year.

### Comparator

The comparator arm (ranibizumab monotherapy) did not receive any planned PRP.

### Assessments

Best-corrected visual acuity (BCVA) was measured at 4 m on a standard Early Treatment Diabetic Retinopathy Study (ETDRS) chart at every visit. OCT was performed on the Heidelberg Spectralis OCT Machine looking at the CRT at every visit. UWFFA was captured on the Optomap P200, using eye steering to ensure the maximum area of retina was imaged.

### Outcome measures

The primary outcome was the number of repeat ranibizumab injections required after the first 6 months up until 1-year post treatment. Secondary outcomes were: the total number of injections given over 1 year; difference in BCVA at 1 year; changes in area of retinal ischaemia on UWFFA; changes in macular thickness (CRT) on OCT.

### Statistical analysis and sample size

The statistical methods and sample size calculations to examine whether the number of repeat injections differed between the PRP and non-PRP groups was conducted based on intention to treat. In order to allow time for any effect of the PRP treatment to become evident, a lag period of 6 months was used; consequently, the number of injections during the final 6 months of the period of observation was analysed.

The average numbers of repeated injections in the PRP and non-PRP groups was compared on the basis that, for any patient the number of injections followed a binomial distribution with a possible maximum equal to the total number of monthly visits during the relevant period (i.e., six during the final 6 months of observation). To allow for the possibility that these numbers were not binomially distributed, a Mann–Whitney test was also used to test for differences between groups. If adjustment is required for other variables (e.g., level of ischaemia), then logistic regression was additionally performed.

In order to have 90% power to detect a difference between groups in the mean number of repeat injections during the final 6 months of the 12-month study period, based on a two-sided test at the 5% level and assuming mean numbers 2.5 and 4 in the PRP and non-PRP groups, respectively, then a total of 28 patients would be required. Alternatively, if the mean numbers were 3 and 4, respectively, then a total of 60 patients would be required. Based on this, we opted for a target of 50 patients.

### Reading centre methodology for measuring retinal ischaemia

The UWFFAs were read retrospectively. The images were sent to the Central Angiographic Resource Facility (CARF), Institute of Clinical Science, Queens University, Belfast and graded for degree of retinopathy and area of ischaemia at baseline and 1 year.

**Fig. 1:**
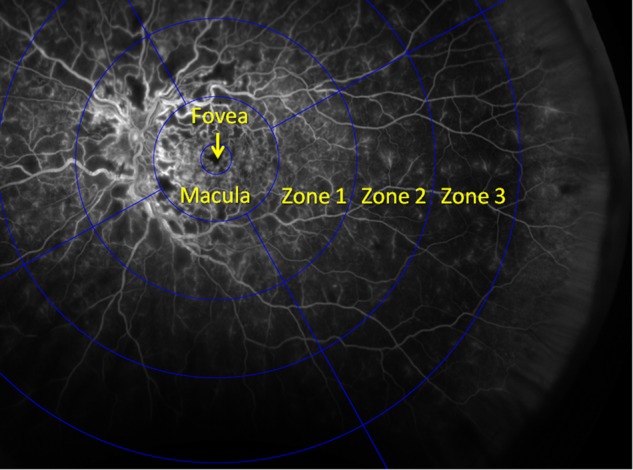
Grid designed and placed on the foveal centre, to measure the area of and change in ischaemia

The method for measuring the area of ischaemia and change in ischaemia involved placing a designed grid centred on the foveal centre with the following zones: Foveal zone: diameter of 1.8 mm ( = 1DD); Macular zone: diameter of 7.2 mm (foveal circle is centralised within this area); Zone 1: diameter of 16.2 mm; Zone 2: diameter of 25.2 mm; Zone 3: diameter of 35.0 mm. This was applied to the UWFFA images using automated software that calibrated the grid to the size of the image being graded, after the foveal centre was manually marked by the grader. The area beyond zone 3 was disregarded as it was found to be incomplete or ungradable in a majority of images (Fig. 1). Ischaemia was graded in the macular zone and zones 1–3. Each zone outside the macula was divided into four sectors—superior, inferior, temporal, and nasal as shown in Fig. 1. Within the macula and each of the 12 sectors, the total area of capillary non-perfusion (CNP) was estimated by an experienced grader and one of the following five grades assigned: no CNP, 1–25%, 26–50%, 51–75%, 76–100%. CNP was defined as an area of the capillary network that fails to fill with fluorescein by the late arteriovenous phase, with minimum linear dimension (MLD) ≥ 63 µm. If > 50% area of a sector was ungradable, then the grade ‘Cannot grade’ was assigned.

A final percentage CNP grade for total retinal non-perfusion was given for the gradable part of the retina by considering the individual grades for all the different zones graded. Areas with PRP laser could not be graded satisfactorily and therefore it was decided to not assign any scores to them. At year 1, images were graded in a similar manner and if new CNP was detected or an increase in CNP was found, this was stated and the retinal quadrant in which this was seen was asked to be specified.

## Results

There were 49 patients recruited from 7 centres, 25 in the ranibizumab only group and 24 in the ranibizumab + PRP group. Eighty-seven percent completed 1-year follow-up. Three patients in the ranibizumab-only arm did not complete the study, one did not want to remain under follow-up and two died. Three did not complete in the combined arm, two did not want to remain under follow-up and one died. The groups were reasonably balanced with a mean baseline age of 63 years, HbA1c of 80 mmol/mol, blood pressure of 143/80 mmHg, OCT CRT of 391 µm, and area of ischaemia of 44%. The mean baseline visual acuity was better in the ranibizumab-only arm at 73 ETDRS letters compared with 67 ETDRS letters (Table 2).

**Table 2:** Patient demographics

	Ranibizumab only	Ranibizumab+PRP	Total	*p*-Values
No. of eyes	25	24	49	N/A
No. of patients	25	24	49	N/A
Right eye % (no/total)	64% (19/25)	50% (12/24)	63% (31/49)	N/A
Age (years), mean (range)	62.64 (40–84)	64.91 (48–83)	63.73 (40–84)	0.4082
Women % (no/total)	16% (4/25)	25% (6/24)	20.41% (10/49)	N/A
HbA1c mean (range)	77.78 (45–113)	82.20 (55–111)	80.11 (45–113)	0.6464
BP (mmHg) (average)	138/80	148/81	143/80	0.1553/0.7778
PDR % (no/total)	8% (2/25)	8.33% (2/24)	8.16% (4/49)	N/A
Beginning BCVA mean (range)	73.68 (48–95)	67.29 (52–89)	70.55 (48–95)	0.0229
Ending BCVA mean (range)	77.88 (56–92)	70.79 (52–89)	74.41 (52–92)	0.0177
Beginning CRT (µm) mean, (range)	378.36 (308–532)	405.67 (301–1023)	391.74 (301–1023)	0.4704
Ending CRT (µm) mean, (range)	316 (224-427)	310.79 (180–541)	313.45 (180–541)	0.7346
Beginning ischaemia (%) mean, (range)	43% (25–75%)	45.45% (0–100%)	44.15% (0–100%)	0.8621
Ending ischaemia (%) mean, (range)	29.55% (0–50%)	39.47% (0–75%)	34.15% (0–75%)	0.2301

*BCVA* best-corrected visual acuity, *BP* blood pressure, *CRT* central retinal subfield thickness,

*ETDRS* Early Treatment Diabetic Retinopathy Study, *HbA1c* glycated haemoglobin, *PDR* proliferative diabetic retinopathy

Both arms had two patients that had mild new vessels detected on the baseline FFA by the reading centre. These patients were also included in the analysis. The average number of injections in the ranibizumab-only arm was 6.84 and the number between 6 and 12 months was 2.52 (SD 2.24). The average number of injections in the combined arm was similar at 6.67, with the number of injections in the second 6 months being 1.92 (SD 2). For the primary outcome, comparing the number of 6- to 12-month injections, the result was not statistically significant (*p* = 0.33).

Similarly comparing the total number of injections in both groups, the differences were not statistically different (*p* = 0.84). The total number of injections also did not relate to the area of ischaemia in either arms or in the arms combined (*p* = 0.24).

There was a statistically significant improvement in visual acuity in each arm, with an average starting BCVA in the ranibizumab-only arm of 73.7 ETDRS letters improving to BCVA 77.8 letters ( + 4 letters) and in the combined arm from BCVA 67.3 letters to BCVA 70.8 letters ( + 3.5). Allowing for the difference in the baseline BCVA measurements, there was no difference in the mean change in the visual acuity between the arms although 84% (21/25) gained vision in the ranibizumab-only arm and only 16/24 (66%) in the combined arm, using last observation carried forward for the three in each arm without final data. This difference was also not significant (*p* = 0.99). Two patients lost >10 letters in the ranibizumab arm only and none in the combined arm. One of these still had very good vision, having started at 95 letters, which dropped to 82. This may have been due to the development of some cataract. The macular oedema had dried up and the retinopathy grade of severe non proliferative diabetic retinopathy (NPDR) was the same at the end of the year. In the second case, the vision dropped from 75 letters to 56. The retinopathy grading remained as severe NPDR. The image quality at the end was worse consistent with cataract development, although there was persistent macular oedema. In the ranibizumab-only arm, 21/25 also had a final visual acuity of over 72 letters compared with 11/24 in the combined arm, although the starting vision was lower in this group.

**Fig. 2:**
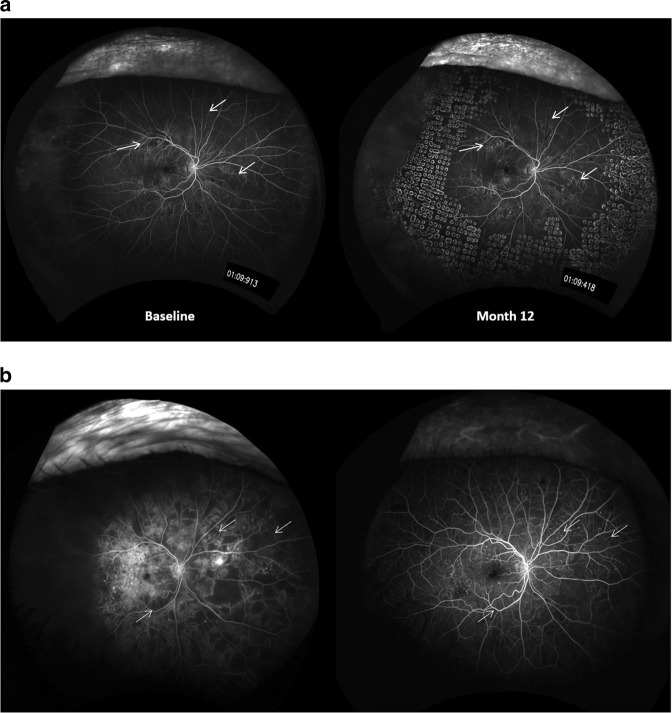
**a** Ranibizumab and laser arm: white arrows showing the areas of change and re-perfusion in the month 12 image compared with baseline. (Images chosen at the closest time point in FFA run). **b** Ranibizumab arm: white arrows showing the areas of change and re-perfusion in the month 12 image compared with baseline. (Images chosen at the closest time point in FFA run)

There was a statistically significant improvement in the area of retinal ischaemia in the ranibizumab-only arm (*p* = 0.0045) compared with baseline but not in the combined arm (*p* = 0.29). Ten improved and one became more ischaemic in the ranibizumab arm despite 10 injections in the patient who became worse. In the combined arm, seven improved and four became more ischaemic, the rest having the same area of ischaemia (Table 3; Figs. 2a, b).

**Table 3:** Comparing the grade of retinopathy and proportion of ischaemia in both groups

	Ranibizumab (*n* = 25)		Ranibizumab + PRP (*n* = 24)	
	Grade of retinopathy	Proportion of ischaemia	Grade of retinopathy	Proportion of ischaemia
No change	18	11	16	9
Improvement	3	10	3	7
Worsening	4	1	4	4
Unknown	0	3	1	4

**Fig. 3:**
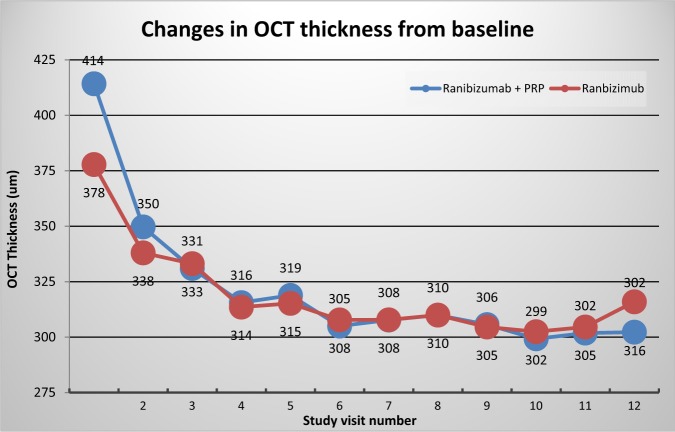
Changes in optical coherence tomography (OCT) thickness from baseline in both the study arms

The OCT thickness decreased by a similar amount in both groups—in the ranibizumab-only arm from 378 µm to 318 µm and in the combined from 405 µm to 310 µm (Fig. 3).

No significant difference was found in either group when comparing the following: OCT thickness at baseline and the area of ischaemia (ranibizumab arm *p* = 0.72, combined arm *p* = 0.17); reduction of OCT thickness at year 1 and the area of ischaemia (ranibizumab arm *p* = 0.15, combined arm *p* = 0.32); total number of injections and initial ischaemia (ranibizumab arm *p* = 0.65, combined arm *p* = 0.28); total number of injections and improvement in ischaemia (ranibizumab arm *p* = 0.79, combined arm *p* = 0.98).

Looking at the OCT thickness at baseline of all the patients (*n* = 49), there was no statistically significant difference when compared with the initial area of retinal ischaemia (*p* = 0.17). The speed of reduction of the OCT thickness did not vary between the two arms (Fig. 3).

In the ranibizumab arm, FFA at 1 year found an additional two patients had developed new vessels and an additional three patients in the combined arm. No additional laser had been required during the study. No patients had any serious ocular adverse events, in particular no endophthalmitis, although two patients lost >10 letters of vision. Nineteen serious adverse events (SAEs) were reported, requiring hospital admission, evenly distributed between the two groups, including three deaths. The deaths were related to heart disease and the other SAEs were largely related to complications of diabetes including kidney disease.

## Discussion

Trials using anti-VEGF therapy show that a high number of treatments are required [1, 2]. In the DRCR.net study Protocol I [1], only a total of 17 eyes (9%) in the ranibizumab + prompt laser group and 15 eyes (8%) in the ranibizumab + deferred laser group met ‘success’ criteria at 16 weeks and did not receive an additional injection before the 1-year primary outcome visit. In the RDP study, the addition of peripheral laser did not reduce the number of injections in total or in the second 6 months. More patients gained vision in the ranibizumab-only arm although a significant difference in the mean vision change was not observed. The mean number of ETDRS letters gained in either arm was less than expected compared with other studies, however, the starting vision was higher and the OCT thickness lower. In the DAVE study [4], there was also a suggestion that the vision improvement was not as good in the combined arm. The number of injections given in the RDP was a little lower than might have been expected in both arms, at 6.7 compared with the median number of 8 injections in the two arms of the DRCR.net Protocol I study, although similar to the mean number of 7 used in the Restore study.

There was some improvement in the overall area of retina measured as perfused in the ranibizumab arm but not in the combined arm. This may not be a fair comparison as the area of retina measured varied as it was not possible to make a clear judgement of perfusion in the areas lasered. Judging changes in areas of perfusion can also be difficult. It maybe that optocal coherence tomography angiography (OCTA) would be better for this but this was not done and currently technology limits the retinal field that can be imaged with OCTA. Neither ranibizumab monotherapy nor the addition of laser prevented the development of new vessels completely. The hypothesis was that treating the non-perfused area of the peripheral retina would reduce the amount of VEGF and so lead to reduced anti-VEGF therapy. Ultra widefield FFA (UWF-FFA) has been shown to better demonstrate the full extent of diabetic retinopathy compared with seven-field imaging [5] but the association of peripheral retinal non-perfusion with diabetic maculopathy is not clear. Sim et al. [6] found an association of peripheral retinal ischaemia with an enlarged foveal avascular zone but not macular oedema, whereas a study by Wessel showed a correlation [3].

In a study of 148 eyes from 76 patients, before the advent of anti-VEGF injections, using UWF-FFA to measure the area of non-perfusion, eyes with larger areas of retinal non-perfusion and worse diabetic retinopathy needed more macular laser [7].

In a study of 99 eyes being treated for DMO with ranibizumab, 49% were associated with microaneurysms, 37% with peripheral ischaemia and 15% with neovascularisation. The oedema associated with peripheral ischaemia was more likely to have a diffuse as opposed to focal pattern and there was a positive correlation of response to the ischaemic index. Whether treating this ischaemia would reduce the recurrence rate was not addressed [8].

Two trials combining UWFFA targeted laser with anti-VEGF treatments suggested that this combination was effective, however, they only had 6 months follow-up and other variables make the conclusions uncertain [9, 10]. The RaScaL trial [10] studied 30 eyes of 22 patients randomised to a combination therapy of ranibizumab plus UWFFA-guided laser compared with a control group that received combination therapy of intravitreal triamcinolone plus focal macular laser. Fewer patients in the ranibizumab plus laser arm needed re-treatment. Takamura et al. [9] randomised 52 patients to bevacizumab or bevacizumab plus targeted PRP then gave all patients focal laser 2 weeks later and monitored the increase in CRT. In the group with the additional targeted PRP they found less increase, and this also related to the area of non-perfusion. Two-thirds of eyes already had already received standard PRP at baseline.

The Relate study [11] and a small study by Spaide [12] found that the addition of PRP to ranibizumab injections for macular oedema due to central retinal vein occlusion did not show a benefit.

In an analysis of the baseline images of the DAVE study [13] involving 40 eyes of 29 patients, randomised to anti-VEGF with or without laser to peripheral ischaemia, the non-perfused area and total retinal area visible was used to calculate an ischaemic index in different retinal zones and correlated to the severity of DMO. The ischaemic index increased with increasing distance from the fovea but the severity of the oedema did not correlate with the overall non-perfused area or ischaemic index [13]. A measure of overall leakage may better relate to VEGF levels but quantifying leak from FFA photographs is difficult as the brightness of an image varies along the run depending on the light exposure.

In the RDP study, one PRP laser of 2000 shots, using the PASCAL laser, was applied to the ischaemic area found on UWF-FFA. This amount was chosen as it was felt it would give some consistency between investigators, would not be too small an amount to have an effect but would be unlikely to risk visual field loss for what was an unproven treatment. This might have limited the benefit of the laser, as the amount could have been varied depending on the amount of ischaemia, however, in the DAVE study [4], heavier titrated laser was applied and repeated during the course of the study. Both approaches did not show an effect. Both studies are small but do not suggest that the addition of laser to ranibizumab monotherapy is beneficial or more precisely the addition of PRP is not more efficient at reducing VEGF levels than anti-VEGF alone. Laser may in fact reduce the visual acuity gains that is achieved with ranibizumab monotherapy. This might be because PRP does not reduce the VEGF levels enough in the vitreous to affect the macula and that the non-perfused area on the FFA is not the only source of cytokines, which promote the development of macular oedema.

Putting the two studies together, it seems most likely that posteriorly produced VEGF or local intra-retinal VEGF and other cytokines at the macula are the main cause of macular oedema.

### Summary

#### What was known before

- Ranibzumab works well for diabetic macular oedema (DMO) but many injections are needed in the first year.
- Ischaemic retina produces VEGF and blocking VEGF reduces macular oedema.
- PRP can reduce VEGF levels.

#### What this study adds

- The addition of PRP to ranibizumab treatment for DMO does not reduce the number of injections required in the first year of ranibizumab therapy.
